# Life-Threatening Complications Associated with Bladder Decompression: A Case Report

**DOI:** 10.5811/cpcem.2022.9.57956

**Published:** 2022-11-04

**Authors:** Barry J. Knapp, Lauren Apgar, Kirstin Pennell

**Affiliations:** Eastern Virginia Medical School, Department of Emergency Medicine, Norfolk, Virginia

**Keywords:** urinary retention, bladder decompression, hematuria, hypotension, case report

## Abstract

**Introduction:**

The clinical course of patients who present to the emergency department (ED) with urinary retention is usually uneventful. In this case, we explore the life-threatening complications of urinary retention and bladder decompression.

**Case Report:**

We report the case of a 57-year-old man who presented to the ED with difficulty voiding. A urinary catheter was placed. The patient had severe post-obstructive diuresis. He developed hematuria and became hypotensive. After aggressive resuscitation, including blood products, the patient required operative intervention for hemorrhage control.

**Conclusion:**

Clinicians should be aware of and be able to manage the rare but life-threatening complications associated with urinary retention.

## INTRODUCTION

Urinary retention is a common problem for men who present to the emergency department (ED). It is estimated that one in three men in their 80s will develop an episode of urinary retention.^1^ Emergency department management is usually straightforward with bladder decompression accomplished after placement of a urinary catheter. Although not common, severe complications associated with urinary retention and bladder decompression can occur. Acute renal failure, electrolyte abnormalities, post-obstructive diuresis, gross hematuria, and hypotension are well documented complications in the literature.

Severe complications associated with urinary retention and bladder decompression are uncommon and usually self-limiting. Reports of patients requiring life-saving interventions are rare. We present the case of a patient with urinary retention who suffered multiple severe complications after bladder decompression requiring aggressive resuscitation and, ultimately, operative intervention. With this report we aim to increase clinician awareness of these uncommon but potentially life-threatening diagnoses.

## CASE REPORT

A 57-year-old man with a history of an unknown previous urologic surgery as a child presented to the ED because of difficulty voiding for approximately 10 days. He reported dribbling when trying to urinate, which later progressed to urinary incontinence. Seven days prior to presentation, he also developed left lower extremity swelling. On ED arrival, he had a blood pressure of 187/111 millimeters of mercury (mm Hg) and a heart rate of 104 beats per minute.

His physical exam was notable for a minimally tender, distended abdomen. The entire left lower extremity was moderately swollen compared to the right, although well-perfused. Routine labs were sent, and the emergency physician performed a point-of-care abdominal ultrasound (POCUS). Normal sonographic abdominal anatomy in the right and left upper quadrants was challenging to identify with several large areas of hypoechoic fluid noted. Owing to the historical concern for urinary retention, bladder catheterization was initiated in the ED. The catheter insertion was atraumatic with an immediate return of one liter of urine. The nurse clamped the catheter to prevent further rapid bladder decompression.

Laboratory studies were notable for an initial creatinine of 12.4 milligrams per deciliter (mg/dL) (reference range: 0.7–1.3 mg/dL), blood urea nitrogen of 77 mg/dL (6–24 mg/dL**)**, potassium of 5.9 milliequivalents per liter (mEq/L) (3.5–5.0 mEq/L), and bicarbonate of 14 mEq/L (23–29 mEq/L). The patient’s initial hemoglobin (Hgb) was 10.1 grams per deciliter (g/dL) (14–18 g/dL). The urinalysis showed 10–20 white blood cells and 50–100 red blood cells (RBC) per high power field.

**Image 1:**
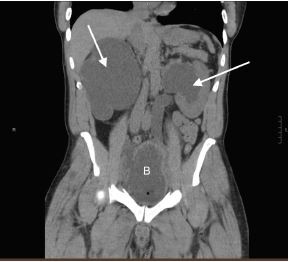
Computed tomography (CT) of the abdomen and pelvis without contrast (coronal view). Note the severe bilateral hydronephrosis (arrows) and bladder distention (indicated by the letter B). The patient had approximately 1,200 milliliters of urine output at the time of the CT.

**Image 2:**
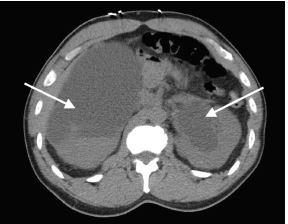
Computed tomography of the abdomen and pelvis without contrast (axial view). Severe bilateral hydronephrosis is seen (arrows). The large areas of fluid are likely responsible for confusing point-of-care ultrasound findings.

A computed tomography (CT) of the abdomen and pelvis was ordered owing to the abnormal initial POCUS (Images 1 and 2). The CT images were acquired after approximately 1,200 milliliters (mL) of urine output from the bladder. The radiologist’s interpretation was: “Severe bilateral obstructive uropathy. Diffuse bladder wall thickening may relate to chronic cystitis, but underlying malignancy is not excluded. Bladder diverticula are noted.”

A urologist was consulted in the ED and requested the bladder catheter remain unclamped. After the clamp was removed, the patient rapidly drained over seven liters of urine. The urine output became grossly bloody during the latter portion of the bladder decompression. During decompression, the patient developed diaphoresis and fatigue. At that time, his blood pressure was found to be 70/52 mm Hg. An intravenous (IV) fluid bolus was initiated. The patient ultimately received a total of three liters of normal saline while in the ED.

Owing to the degree of hematuria, serial Hgbs were sent during his ED and hospital stays. Eight and one-half hours after the initial presentation, the patient’s Hgb dropped to 5.7 mg/dL, a drop in 4.4 mg/dL from the initial level. Additional volume resuscitation was initiated with packed RBCs. A total of four units of packed RBCs were transfused. The patient was admitted in the intensive care unit and treated further with continuous bladder irrigation (CBI).

**CPC-EM Capsule**

What do we already know about this clinical entity?

*Complications of bladder decompression for patients experiencing urinary retention include hemorrhage and post-obstructive diuresis. These complications are usually self-limiting*.

What makes this presentation of disease reportable?

*We report the rare case of a 57-year-old man with post-decompression hemorrhage, diuresis and hemodynamic collapse requiring aggressive resuscitation and operative intervention*.

What is the major learning point?

*Life-threatening complications associated with bladder decompression are rare though can occur*.

How might this improve emergency medicine practice?

*Awareness of the potentially severe complications associated with bladder decompression will allow the clinician to anticipate and intervene quickly when they do occur*.

Owing to continued hemodynamic instability and hemorrhage, the patient required operative intervention. He underwent cystoscopy, bladder biopsy, clot evacuation, and fulguration. Postoperatively the patient had continued bladder hemorrhage and was treated with tranexamic acid administered through the urinary catheter along with steroid infused-CBI.

The patient’s course was complicated by the recognition of bilateral deep vein thrombosis (DVT) while in the intensive care unit. He was started on IV heparin. He subsequently redeveloped gross hematuria and required further blood transfusion. Additionally, the patient underwent bilateral percutaneous nephrostomy (PCN) placement to divert urine away from the bladder. The patient was eventually discharged on day five with an inferior vena cava filter and bilateral PCN. Upon discharge, the patient’s creatinine was 1.8 mg/dL. The cause of the patient’s initial urinary retention was never definitively determined.

## DISCUSSION

The presentation of acute urinary retention is usually straightforward—severe abdominal pain associated with the inability to void. Clinicians must be aware that the presentation of chronic urinary retention can be more insidious. As was the case in our patient, abdominal pain may not be a historical feature. Emergency physicians need to recognize urinary retention in its various forms and understand the uncommon though serious complications that can occur.

The normal bladder can hold 450–500 milliliters (mL) of urine. An obstruction of urinary outflow can be precipitate acute renal failure. Our patient presented in acute renal failure with an initial creatinine of 12.4 mg/dL. Fortunately, the patient’s potassium was not severely elevated (5.9 mEq/dL). In most cases of post-obstructive renal failure, relieving the obstruction will enable the return of baseline renal function. The patient’s creatine returned to its baseline level during his hospital course without the need for dialysis.

Our patient decompressed over seven liters of urine during the first five hours of his ED stay. Patients who put out more than 1,500 mLs of urine immediately after bladder catheterization are thought to be at higher risk of developing post-obstructive diuresis,^2^ which is seen in up to 52% of patients with urinary retention.^3^ It is defined as an output of ≥200 mLs of urine for ≥two hours after the initial decompression, or greater than 3,000 mLs in the first 24 hours.^2^ Post-obstructive diuresis is primarily a problem with chronic, not acute, urinary retention and usually represents an appropriate attempt to excrete excess fluid retained during the period of obstruction.^4^

Although rare, post-obstructive diuresis can lead to hypotension and hemodynamic collapse. This was the case for our patient. Volume replacement should be initiated early in these patients with a recommendation that no more than 75% of the average hourly urine output be replaced to avoid stimulation for further diuresis.^5^

Hematuria is an additional recognized complication of bladder decompression. Hematuria is reported to occur in 2–16% of patients.^6^ The mechanism of hemorrhage post-decompression is not clearly understood, although it is thought to be related to bladder stretch injury. Bladder hemorrhage is usually self-limited and rarely requires aggressive or invasive intervention. It was previously thought that complications associated with bladder decompression could be avoided by gradually releasing urine over a period of hours. More recent literature supports the practice of rapid bladder decompression as it has not been shown to increase complication rates.

Etafy et al randomized two groups with acute urinary retention to receive either rapid or gradual bladder decompression. Of the 31 patients in each cohort, no significant complications in either group were noted.^7^ Similarly, Boettcher et al randomized 294 patients into rapid and gradual decompression groups. Their study differed in that it included patients with both acute and chronic urinary retention. They found no statistically significant difference in complication rates between gradual and rapid bladder emptying. They concluded that gradual emptying did not reduce the risk of hematuria or circulatory collapse and that there is no need to prefer gradual over rapid emptying.^8^

Although they may not be influenced by the rate of bladder decompression, complications including hematuria, post-obstructive diuresis, and hypotension do occur. When these complications happen, they are rarely clinically significant.^3^ When they are, clinicians must be ready to intervene. Owing to persistent hemorrhage and hemodynamic instability despite IV fluids, our patient required transfusion of multiple units of packed RBCs. As his bladder hemorrhage was not self-limited, hemorrhage control in the operating room by the urologist was required.

Our patient was subsequently diagnosed with bilateral DVTs. An interesting association between urinary retention and DVT exists. Lower extremity clot formation is likely related to direct compression of the iliac veins, which creates stasis in the venous system. Deep vein thrombosis associated with urinary retention is rare, although it has been reported in the literature.^9^ The venous stasis and resulting thrombotic complications caused by urinary retention should prompt clinicians to perform a detailed physical examination, including the identification of leg swelling. For patients with associated shortness of breath, an investigation for pulmonary embolism should be undertaken. The anticoagulation required for the treatment of an acute DVT further complicated the management of our patient’s bladder hemorrhage.

Identifying patients who will develop severe complications after bladder decompression is not straightforward. Patients who develop persistent hypotension and gross hematuria will benefit from urologic consultation and hospital admission. As chronic urinary retention with high volumes of urine output (greater than 1,500 mLs) are at greater risk for post-obstructive diuresis, it is reasonable to observe those patients for 24 hours in the hospital setting. For others, it is recommended that patients be monitored for a minimum of four hours for significant hourly urinary output (>200 mL per hour over intake) after the initial return. If this degree of output continues, the patient should be admitted with appropriate volume replacement.^1^

## CONCLUSION

Understanding the complications associated with urinary retention and bladder decompression is essential for clinicians. Emergency physicians must ensure they are adequately prepared to recognize and manage these rare but potentially life-threatening problems.
